# Spatial distribution of parrotfishes and groupers in an Okinawan coral reef: size-related associations in relation to habitat characteristics

**DOI:** 10.7717/peerj.12134

**Published:** 2021-09-03

**Authors:** Atsushi Nanami

**Affiliations:** Yaeyama Field Station, Coastal and Inland Fisheries Ecosystem Division, Environment and Fisheries Applied Techniques Research Department, Fisheries Technology Institute, Japan Fisheries Research and Education Agency, Ishigaki, Okinawa, Japan

**Keywords:** Parrotfishes, Groupers, Spatial distribution, Coral reef, Habitat association, Habitat characteristics, Species-specific distribution, Size variation

## Abstract

Parrotfishes (Labridae: Scarini) and groupers (Epinephelidae) are important fish groups that are regarded as the fisheries targets of primary importance in coral reefs. In order to establish ecosystem-based management of these two fish groups, clarifying the spatial distribution relative to habitat characteristics is of central importance. The present study investigated the spatial distributions of 12 parrotfishes species and seven groupers species in relation to environmental characteristics in an Okinawan coral reef. Ten out of the 12 parrotfish species and all seven grouper species showed species-specific spatial distributions. Four substrate types in the inner reefs (branching *Acropora*, bottlebrush *Acropora*, dead branching *Acropora*, and dead bottlebrush *Acropora*), three substrate types in the exposed reefs (massive coral, other coral, and calcium carbonate substratum), and water depth showed significant associations with the spatial distribution of fishes. Among the 12 parrotfish species, two species (*Scarus spinus* and *S. forsteni*) and four species (*S. psittacus*, *S. hypselopterus*, *S. dimidiatus* and *S. ghobban*) were primarily found in exposed reefs and inner reefs, respectively. Among the seven grouper species, two species (*Cephalopholis argus* and *C. urodeta*) and two other species (*C. miniata* and *Epinephelus ongus*) were primarily found in exposed reefs and inner reefs, respectively. Size-related spatial distribution was also found for three parrotfish species (*Chlorurus microrhinos*, *Scarus rivulatus* and *S. hypselopterus*), indicating that smaller-sized and larger-sized individuals were respectively found at sites with greater coverage of substrates with fine structure (live bottlebrush *Acropora* and dead bottlebrush *Acropora*) and coarse structure (live branching *Acropora*, dead branching *Acropora* and calcium carbonate substratum). The present study suggested that the spatial distribution of parrotfishes and groupers is not necessarily associated with the higher coverage of living corals, but positively associated with high substrate complexity. Thus, actual spatial distributional patterns of species should be considered to select candidate sites for protection and conservation for the two fish groups.

## Introduction

Coral reef ecosystems support a high species diversity of marine organisms. Such a high species diversity provides various ecosystem services such as natural food production, ornamental resources, aquarium resources, habitat maintenance, and recreation (Moberg & Folke, 1999; Laurans et al., 2013; Elliff & Kikuchi, 2017). Among the diverse ecosystem services, the provision of fisheries targets has been recognized as an essential service for coastal communities (Elliff & Kikuchi, 2017; Woodhead et al., 2019). The management of fisheries targets in coral reef areas has been a considerable challenges for many years (Medly, Gaudian & Wells, 1993; Frisch et al., 2016).

Besides achieving effective fisheries management, ecosystem-based management such as habitat conservation and protection of the target species has been the focus of research recently (*e.g*., Fenner, 2012; Frisch et al., 2016; Harvey et al., 2018). Among the ecosystem-based management tools, marine protected areas (MPAs) have garnered considerable attention and have been regarded as an essential tool (Russ, 2002; Fenner, 2012). This is because MPAs can achieve the protection of various sizes of fisheries targets including juveniles and adults as well as conservation of their habitats (Russ, 2002; Sobel & Dahlgren, 2004).

Some previous studies have attempted to protect coral-rich sites and/or restore coral assemblages that are susceptible to coral bleaching and crown-of-thorns starfish outbreaks (*e.g*., Barton, Willis & Hutson, 2015; Lirman & Schopmeyer, 2016; Zayasu & Suzuki, 2019). This is because live corals mainly provide refuge spaces and shelters for fishes and loss of live corals is considered to cause a decline in species diversity (reviewed in Pratchett et al. (2008)). However, some fish species also use non-coralline substrates as their habitats, shelter, and foraging sites (*e.g*., Light & Jones, 1997; Nanami & Yamada, 2008; Nanami et al., 2013; Kramer, Bellwood & Bellwood, 2014, 2016). Thus, coral coverage alone is not necessarily a good indicator for selecting candidate sites for protecting the fish abundance. To select sites for restoration and conservation of fish abundance and species diversity, it is necessary to clarify the actual spatial distribution of the target species. In addition, since Green et al. (2015) suggested that MPAs should be established to protect all life stages of fish (*e.g*., newly settled juveniles, non-adult fishes and adults), clarifying the substrate types providing suitable habitats for size-related fish spatial distributions is also important.

In addition, clarifying the habitat characteristics that are associated with the spatial distribution of target species is important to establish effective MPAs settings (Roos et al., 2020). Numerous studies have suggested that habitat characteristics (*e.g*., coral morphology, coral coverage, and wave exposure) and inter-specific interactions are the main factors that influence both species- and size-specific spatial distributions of coral reef fish (*e.g*., Williams, 1991; Fulton, Bellwood & Wainwright, 2001; Friedlander et al., 2003). For example, it has been suggested that live corals are a determinant of the spatial distribution of various coral reef fish species, because live corals offer food and shelter (*e.g*., Munday, Jones & Caley, 1997; Pratchett, Graham & Cole, 2013; Nanami, 2020). Munday, Jones & Caley (1997) demonstrated clear associations between coral-dwelling gobies and acroporid corals whereas Pratchett, Graham & Cole (2013) and Nanami (2020) showed that live corals were selected as foraging substrate for butterflyfish species. Furthermore, abiotic factors (*e.g*., topographic complexity and wave exposure) have also significant effects on the spatial distribution of fish (*e.g*., Luckhurst & Luckhurst, 1978; McCormick, 1994; Fulton & Bellwood, 2002; Nanami et al., 2005). It is suggested that higher substrate topographic complexity supports greater abundance and species richness of coral reef fishes (Luckhurst & Luckhurst, 1978; McCormick, 1994), whereas the degree of wave exposure affects swimming ability resulting in spatial variations in coral reef fish assemblages (Fulton & Bellwood, 2002).

Among the diverse fish groups in coral reefs, parrotfishes (Labridae: Scarini) are regarded as fishery targets of primary importance (*e.g*., Aswani & Sabetian, 2010; Howard, Schumacher & Parrish, 2009; Mumby, 2016). Some previous studies have examined the zonational spatial distribution of parrotfish assemblages (*e.g*., Cheal et al., 2012; Hoey & Bellwood, 2008; Johnson et al., 2019). These studies have shown cross-shelf variations (inshore, mid-shelf and outer shelf) as well as latitudinal variations in parrotfish assemblages. However, long-term overfishing has caused a severe decline of parrotfish abundance and appropriate management measures for protection required worldwide (*e.g*., Hamilton et al., 2019; Pinheiro et al., 2021).

Groupers (Epinephelidae) are also considered as essential fishery targets in coral reefs (*e.g*., Levin & Grimes, 2002; Howard, Schumacher & Parrish, 2009; Frisch et al., 2016). Newman, Williams & Russ (1997) has shown zonational variations (reef slope, lagoon and back reef) as well as cross-shelf variations (mid-shelf *vs*. outer shelf) in grouper assemblages in the Great Barrier Reef. Since groupers are vulnerable to overfishing (Levin & Grimes, 2002), MPAs are regarded as an effective tool to recover grouper populations (*e.g*. Anderson et al., 2014; Stafford et al., 2016). However, although MPAs should be set to include the key habitats for ontogenetic stages of target fish species (Dahlgren et al., 2006), few previous studies have studied the size-specific variations in spatial distribution of parrotfish and grouper assemblages in relation to various habitat characteristics including live corals, dead corals and non-coralline substrates.

As these two fish groups are the main fisheries targets in the coral reefs of Okinawa, effective conservational actions are required (Akita et al., 2016). However, detailed ecosystem-based management measures for these two fish groups have not been established in this region. To establish effective ecosystem-based management tools, the spatial distribution of the target species in relation to environmental characteristics should be investigated in this region. Thus, the aim of the present study was to investigate the spatial distribution of parrotfishes and groupers on a coral reef in Okinawa. Size-related variations in the spatial distributions were also investigated, and what types of substrate should be focused on to establish effective ecosystem-based management of these two fish groups.

## Materials and Methods

### Data collection of fish and environmental variables

**Figure 1:**
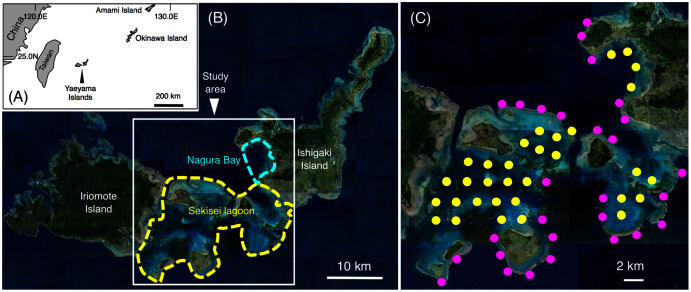
Study site. The maps show the location of the Yaeyama Islands (A), Sekisei Lagoon, and Nagura Bay (B), the 60 study sites used for underwater observations of spatial distribution (C). In (C), magenta symbols and yellow symbols indicate the sites in exposed reefs and inner reefs, respectively. The aerial photographs used in (B) and (C) were provided by the International Coral Reef Research and Monitoring Center.

This study was conducted in Sekisei Lagoon and Nagura Bay, Yaeyama Islands, Okinawa (Figs. 1A, 1B). Sixty study sites were established, and underwater observations were conducted during two periods: (1) between June 2016 and January 2017 and (2) between June 2017 and February 2018. Total number of surveys was 18 days (average intervals = 13.9 days; range = 1–54 days) and 20 days (average intervals = 11.4 days; range = 1–57 days) in the first and second series of observations, respectively. The distance between two sites was approximately 2 km. Among the 60 sites, 28 sites were located in the exposed reefs, and remaining 32 sites were located in the inner reefs (Fig. 1C).

Underwater observations were performed according to Nanami (2018), who provided details of the methods of underwater observations and fish count, and measurement of environmental variables. A 20-min time transect was set (transect width = 5 m) in each site during the day (830–1,600 h). The number of individuals and their total length (TL) were recorded by scuba diving. The distance of each time transect was recorded using a portable GPS receiver. The average distance of the 20-min time transects was 347.4 ± 46.5 m (mean ± standard deviation (SD), *n* = 60). Water depth was recorded using a diving computer (ranged from 3.1 to 11.8 m).

To evaluate substrate availability in each site, digital video images (moving pictures) of the substrate were recorded. Static images were then obtained at 10-s intervals using QuickTime Player Pro software (version 7.6). As a result, 121 static images were obtained per 20-min video image (from 0 to 1,200 s). The substrate at the center of each static image (single pixel) on the monitor of a personal computer was recorded for analysis. For each transect, 121 substrate data (*i.e*., 121 static images) were pooled and regarded as the substrate coverage. The substrate was divided into 16 types for analysis (Fig. S1), following Nanami (2018): (1) branching *Acropora*, (2) tabular *Acropora*, (3) bottlebrush *Acropora*, (4) branching corals except for *Acropora* (*e.g*., branching *Pocillopora*, *Montipora*, and *Porites*), (5) massive corals (*e.g*., massive *Porites* and Faviidae members), (6) other live corals (*e.g*., encrusting corals and foliose corals), (7) dead branching *Acropora* (8) dead tabular *Acropora* (9) dead bottlebrush *Acropora*, (10) dead branching corals, (11) dead other corals, (12) soft corals, (13) coral rubble, (14) calcium carbonate substratum, (15) sand, and (16) macroalgae (*e.g*., *Padina minor* and *Sargassum* spp.). This classification of substrates was also based on Pratchett et al. (2015), which detailed the major growth forms of corals.

### Spatial distribution analysis

Underwater observations revealed that 12 parrotfish species (*Chlorurus bowersi*, *C. spilurus*, *C. microrhinos*, *Scarus dimidiatus*, *S. forsteni*, *S. ghobban*, *S. hypselopterus*, *S. niger*, *S. psittacus*, *S. rivulatus*, *S. schlegeli*, and *S. spinus*) and seven grouper species (*Plectropomus leopardus*, *Variola louti*, *V. albimarginata*, *Cephalopholis argus*, *C. urodeta*, *C. miniata*, and *Epinephelus ongus*) were the dominant species across all 60 sites (Fig. S2). Thus, these species were selected for analyses in this study. The number of individuals was converted into density (number of individuals per 100 m distance × 5 m wide) using the distance data as follows:

$$\matrix{ {\rm{Fish \;density\; at\; each\; site\; per\; 100\; m\; distance }} \cr {\rm{ = [number \;of\; individuals\; at\; each\; time\; transect\; (5\; m\; wide)]/}} \cr {\rm{[distance\; of\; the\; each\; time\; transect\; (5\; m\; wide)] }} \times {\rm{ }}100 }$$

Since it is suggested that MPAs should be set on key habitats for the entire life stage of the target fish species, various growth stages (*e.g*., newly settled juveniles, late-stage juveniles, young fishes and adult fishes) should be considered. Thus, individual fish were categorized into six size classes: class 1 (TL ≤ 10 cm), class 2 (11 cm ≤ TL ≤ 20 cm), class 3 (21 cm ≤ TL ≤ 30 cm), class 4 (31 cm ≤ TL ≤ 40 cm), class 5 (41 cm ≤ TL ≤ 50 cm), and class 6 (TL ≥ 51 cm). To visualize the actual spatial distributions of each species and each size class, pie charts were made on the study map. In these charts, the circle sizes represents the density.

The relationship between the spatial distribution of each size class individuals of the two fish groups and seventeen environmental characteristics (16 substrates types and depth) was analyzed as follows: (1) the species response (linear or unimodal) against the environmental variables were examined by using detrended correspondence analysis (DCA) in accordance with the method detailed in Ter Braak & Smilauer (2002). As a result, DCA revealed linear responses of species against environmental variables; (2) since the relationship between species and environmental variables can be assumed as linear, redundancy analysis (RDA) was conducted to clarify the relationship with CANOCO software (Ter Braak & Smilauer, 2002). This analysis was performed using averaged data from the two series of observations. Before the analysis, the fish density data were log (*x* + 1) transformed. Software options for forward selection were applied to extract the environmental characteristics that significantly affected the spatial distribution.

The interpretations of RDA are as follows: (1) RDA score plotting consists of four quadrants along with two axes (RDA axis 1 and 2); (2) scores of 17 environmental variables (16 substrate types and depth) were shown as vectors; (3) species scores for 19 fish species (12 parrotfish species and 7 grouper species) were shown as points; (4) the relationship between spatial distribution of fish species and environmental variables can be interpreted by the positional relationships between species scores and vectors. Namely, if a species score is plotted near a particular vector, it can be interpreted that there is a close association between the species and the environmental variable; (5) species scores that are apart from the origin of the coordinates (0, 0) represent greater tendency of species-specific or size-specific spatial distribution with a particular environmental variable.

## Results

### Spatial distribution of fish in relation to environmental variables

**Figure 2:**
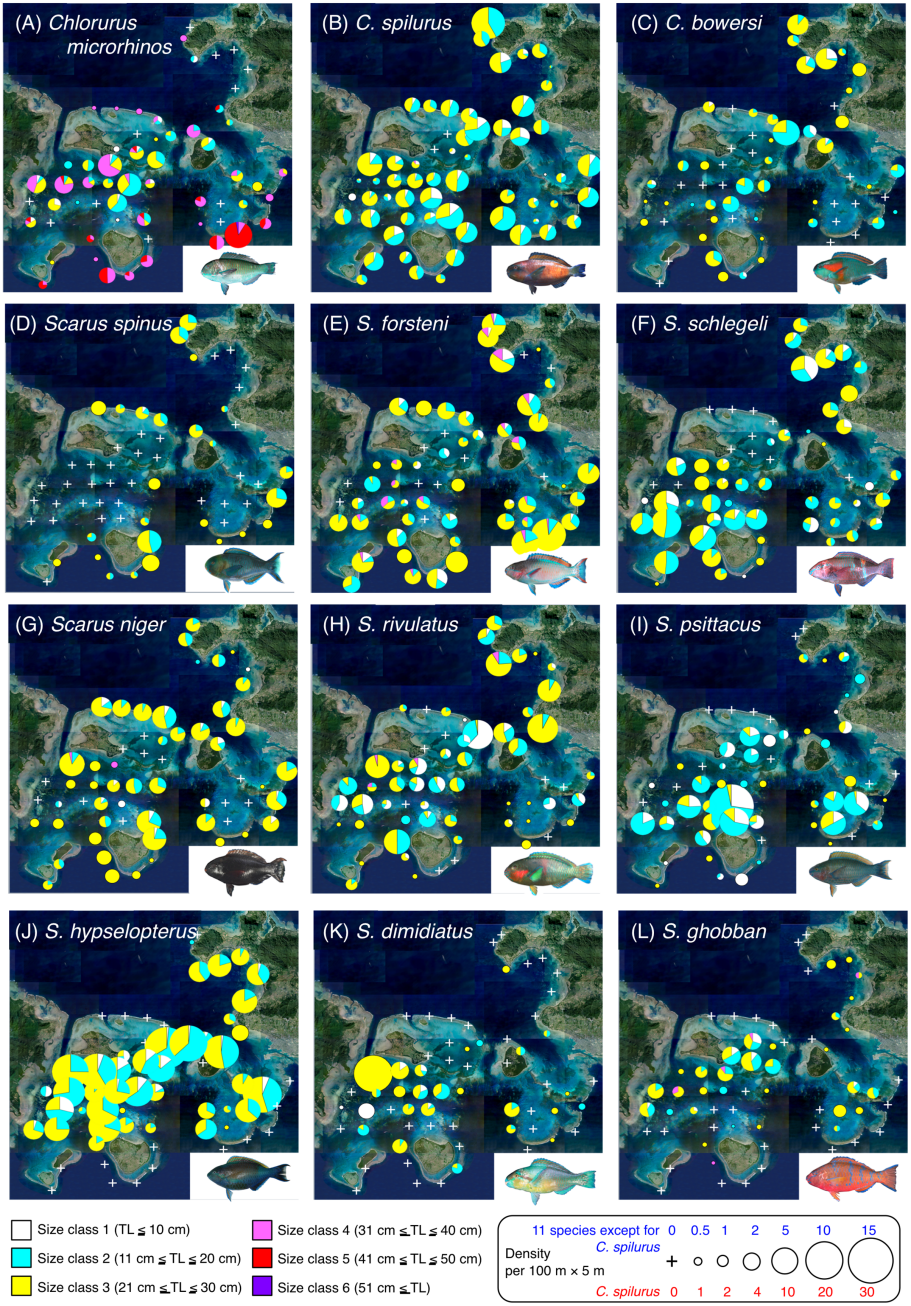
Spatial distributions of the 12 parrotfish species. Six different colors (white, sky-blue, yellow, magenta, red, and purple) and circle diameter represent different size classes and density per 100 × 5 m, respectively. The aerial photographs were provided by the International Coral Reef Research and Monitoring Center. The photographs of all fish species were taken by the author (A. Nanami).

**Figure 3:**
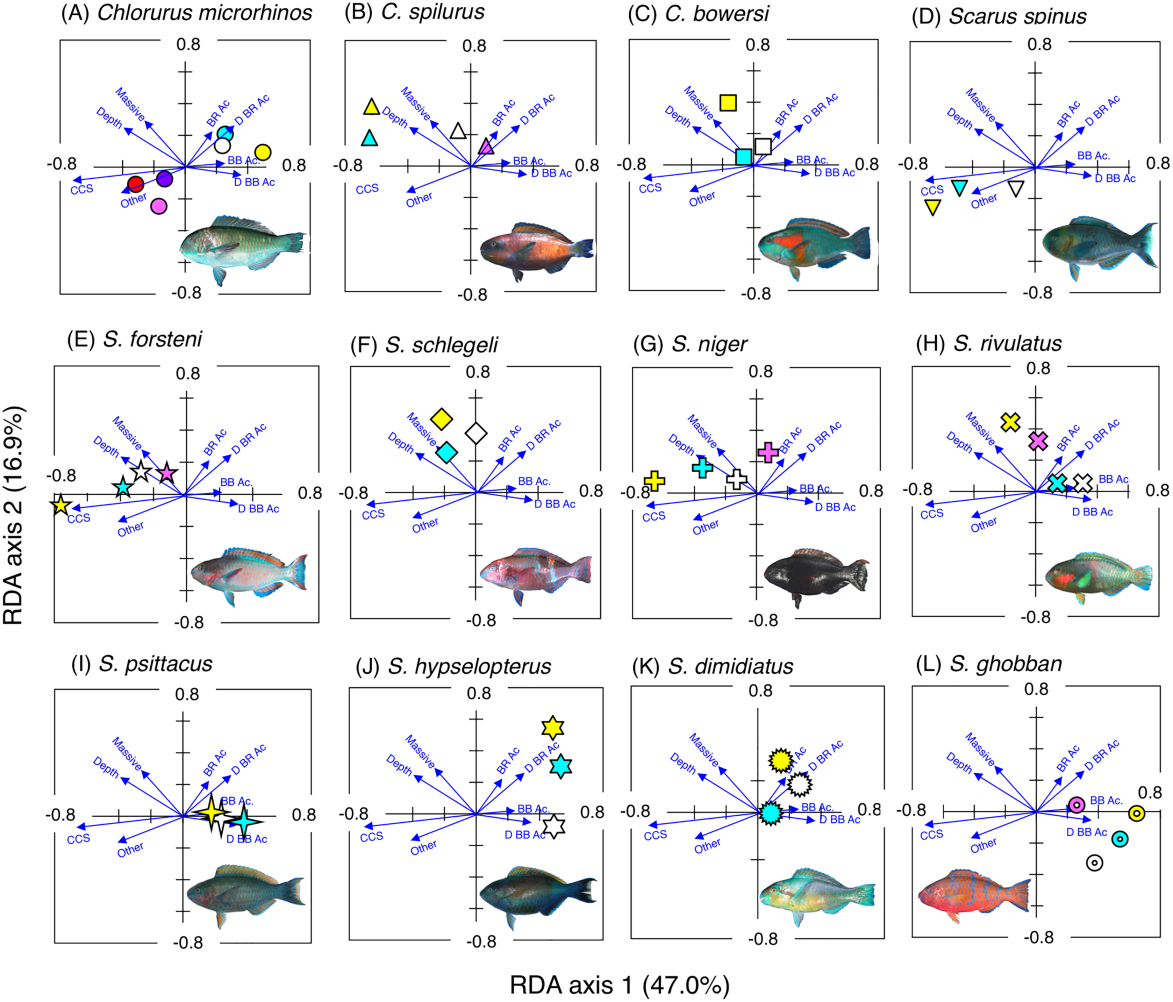
Results of the redundancy analysis (RDA) to explain the association between the spatial distribution of the 12 parrotfish species and environmental characteristics. The result of each species is separately shown for ease of viewing, although the assemblage-level analysis was done (see Fig. S3). Environmental characteristics that had a significant association on spatial distribution are presented as blue vectors (see also Fig. S5). Six different colors of symbols (white, sky-blue, yellow, magenta, red, and purple) represent different size classes (see Fig. 2). Some types of substrates are represented with abbreviations (BR Ac: branching *Acropora*; D BR Ac: dead branching *Acropora*; BB Ac: bottlebrush *Acropora*, D BB Ac: dead bottlebrush *Acropora*, CCS: calcium carbonate substratum). The photograph of all fish species were taken by the author (A. Nanami).

**Figure 4:**
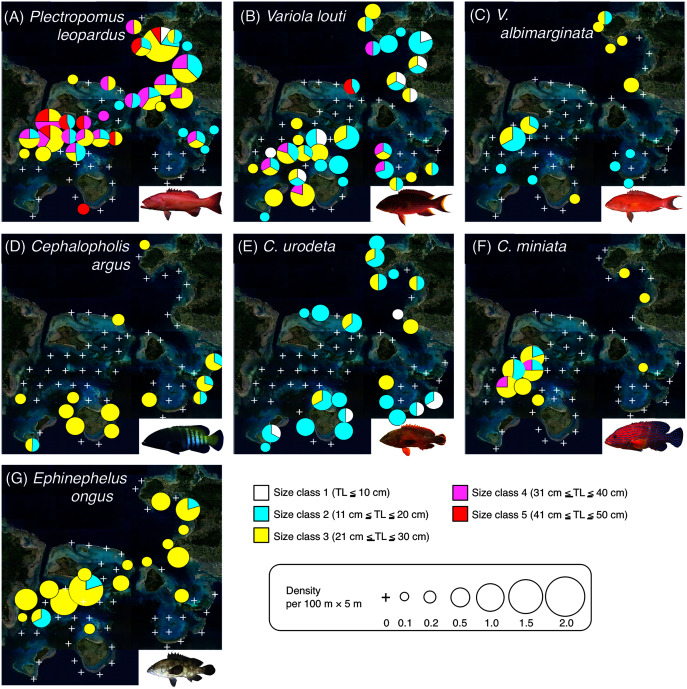
Spatial distributions of the seven grouper species. Five different colors (white, sky-blue, yellow, magenta, and red) and circle diameter represent different size classes and density per 100 × 5 m, respectively. The aerial photographs were provided by the International Coral Reef Research and Monitoring Center. The photograph of all fish species were taken by the author (A. Nanami).

**Figure 5:**
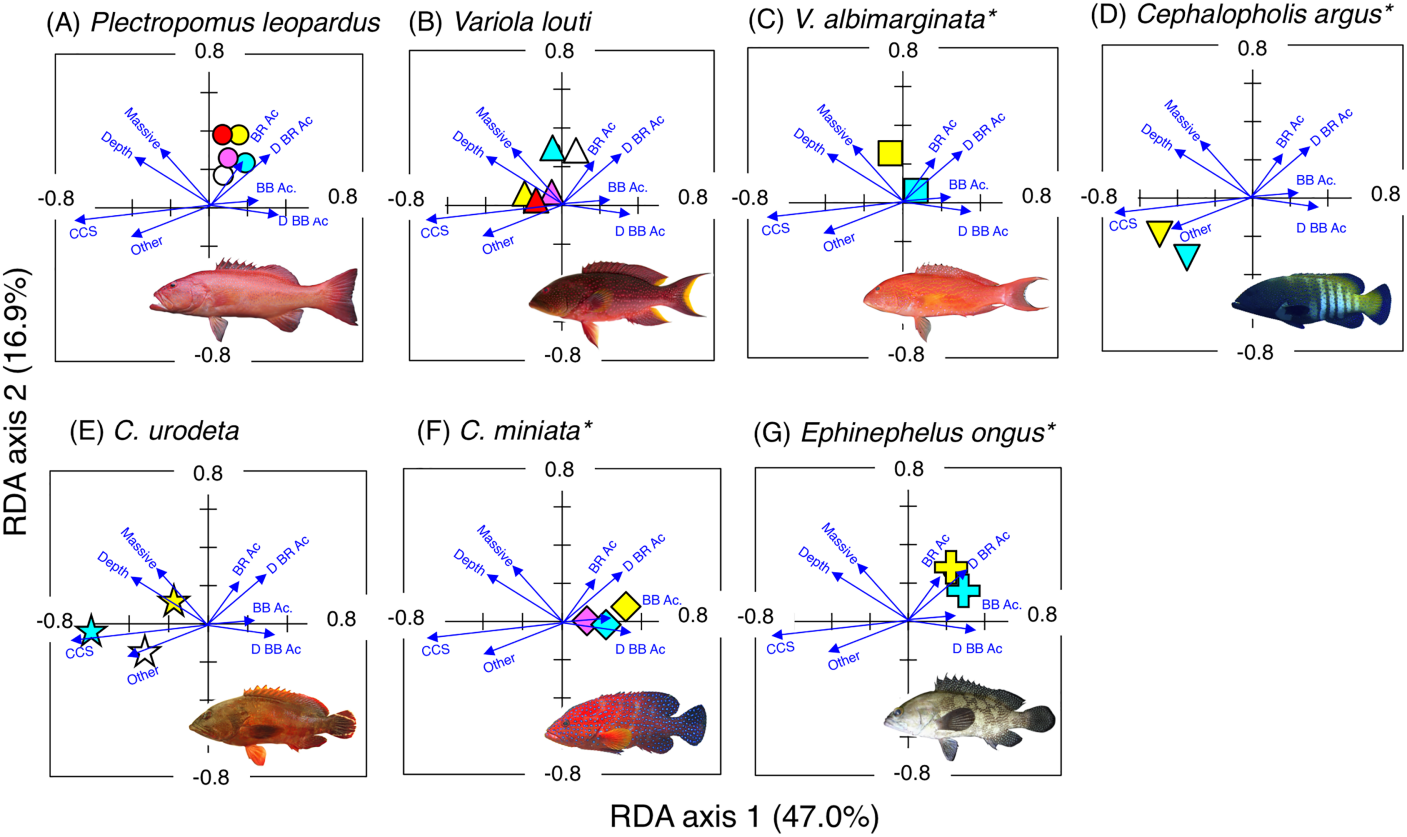
Results of the redundancy analysis (RDA) to explain the association between the spatial distribution of the seven grouper species and environmental characteristics. The result of each species is separately shown for ease of viewing, although the assemblage-level analysis was done (see Fig. S4). Environmental characteristics that had a significant association on spatial distribution are presented as blue vectors (see also Fig. S5). Five different colors of symbols (white, sky-blue, yellow, magenta, and red) represent different size classes (see Fig. 4). Some types of substrates are represented with abbreviations (BR Ac: branching *Acropora*; D BR Ac: dead branching *Acropora*; BB Ac: bottlebrush *Acropora*, D BB Ac: dead bottlebrush *Acropora*, CCS: calcium carbonate substratum). *: No individuals of size class 1 were found for three species (*Variola louti*, *V. albimarginata* and *Epinephelus ongus*). The photographs of all fish species were taken by the author (A. Nanami).

Pie charts and results of RDA showed species-specific spatial distribution of the two fish groups (Figs. 2–5, Figs. S3, S4). The RDA results also revealed the differences in habitat characteristics between the exposed and inner reefs (Fig. S5). The exposed reefs were characterized by a greater coverage of calcium carbonate substratum, massive corals, and other corals, and greater depth. In contrast, the inner reefs were characterized by a greater coverage of branching *Acropora*, bottlebrush *Acropora*, massive corals, other live corals, dead branching *Acropora*, dead bottlebrush *Acropora*, coral rubble, sand, and macroalgae. Among the 17 environmental variables, seven substrate types (branching *Acropora*, bottlebrush *Acropora*, massive corals, other live corals, dead branching *Acropora*, dead bottlebrush *Acropora*, and calcium carbonate substratum) and depth showed significant associations with the spatial distribution of fishes.

### Spatial distribution of parrotfishes

*Chlorurus microrhinos* (Figs. 2A, 3A): Size class 1, 2, and 3 individuals were found in the inner reefs with a high coverage of branching *Acropora*, bottlebrush *Acropora*, dead branching *Acropora*, and dead bottlebrush *Acropora*. Although size class 4 individuals were found in both exposed and inner reefs (Fig. 2A), the overall trend analyzed by RDA revealed that greater density of size class 4 individuals tended to be found in the exposed reef with a higher coverage of calcium carbonate substratum and other corals (Fig. 3A). Size class 5 and 6 individuals were primarily found in the exposed reefs with a high coverage of calcium carbonate substratum and other corals.

*Chlorurus spilurus* and *S. niger* (Figs. 2B, 2G, 3B, 3G): Size class 1 individuals were found in both exposed and inner reefs, whereas size class 2 and 3 individuals tended to be found in the exposed reefs with a high coverage of calcium carbonate substratum. Size class 4 individuals were found in the inner reefs with a high coverage of branching *Acropora* and dead branching *Acropora*.

*Chlorurus bowersi* and *S. schlegeli* (Figs. 2C, 2F, 3C, 3F): The individuals of the three size classes did not show a clear trend of distribution.

*Scarus spinus* and *S. forsteni* (Figs. 2D, 2E, 3D, 3E): The individuals of all size classes were primarily found in the exposed reefs with a higher coverage of calcium carbonate substratum and other corals.

*Scarus rivulatus* (Figs. 2H, 3H): Size class 1 and 2 individuals tended to be found in the inner reefs with a higher coverage of bottlebrush *Acropora* and dead bottlebrush *Acropora*, whereas size class 3 and 4 individuals were found in both exposed and inner reefs.

*Scarus psittacus*, *S. hypselopterus*, *S. dimidiatus*, and *S. ghobban* (Figs. 2I–2L, Figs. 3I–3L): All size class individuals were found in the inner reefs with a greater coverage of bottlebrush *Acropora*, branching *Acropora*, dead bottlebrush *Acropora*, and dead branching *Acropora*.

### Spatial distribution of groupers

*Plectropomus leopardus* (Figs. 4A, 5A): All size class individuals were found in the inner reefs with a higher coverage of bottlebrush *Acropora*, branching *Acropora*, dead branching *Acropora*, and dead bottlebrush *Acropora*.

*Variola louti* (Figs. 4B, 5B): Size class 1 and 2 individuals were found in both exposed and inner reefs with a higher coverage of branching *Acropora* and massive corals. Size class 3, 4, and 5 individuals tended to be found in the exposed reefs with a greater coverage of calcium carbonate substratum.

*Variola albimarginata* (Figs. 4C, 5C): Size class 2 individuals tended to be found in the inner reefs with a higher coverage of bottlebrush *Acropora*, whereas size class 3 individuals were found in both exposed and inner reefs with a higher coverage of branching *Acropora* and massive corals.

*Cephalopholis argu*s and *C. urodeta* (Figs. 4D, 4E, 5D, 5E): All size class individuals were found in the exposed reefs with a higher coverage of calcium carbonate substratum and other corals.

*Cephalopholis miniata* and *E. ongus* (Figs. 4F, 4G, 5F, 5G): All size class individuals were found in the inner reefs with a higher coverage of bottlebrush *Acropora*, branching *Acropora*, dead branching *Acropora*, and dead bottlebrush *Acropora*.

## Discussion

### Spatial distribution of parrotfishes

Previous studies have reported the zonational patterns in parrotfish abundance in relation to topographic features such as reef slope, reef crest, reef flat, and back reef in the Great Barrier Reef and Meso-American Barrier Reef System (*e.g*., Russ, 1984a, 1984b; Gust, Choat & McCormick, 2001; Hoey & Bellwood, 2008; Hernández-Landa et al., 2014). These studies have shown distinct structures for parrotfish assemblages among different zones (*e.g*., exposed reefs *vs*. sheltered reefs). In contrast, the present study examined the associations between the spatial distribution of parrotfishes and substrate characteristics. The results showed that various types of substrates (live corals, dead corals and non-coralline substrates) showed significant associations with the spatial distributions. In the inner reefs, branching *Acropora* and bottlebrush *Acropora* as well as dead colonies of the two types of corals showed significant associations with the spatial distribution. These results suggest that parrotfish species in the inner reefs were positively associated with substrates with a fine structure and greater complexity regardless of whether the substrates are live corals or dead corals. In contrast, in the exposed reefs, calcium carbonate substratum and some live corals (*e.g*., massive corals and other corals (encrusting and foliose corals)) showed significant associations with the spatial distribution. Calcium carbonate substratum inherently possesses a high structural complexity (*e.g*., uneven surfaces and large holes). This suggests that calcium carbonate substratum, which have less complex structures than branching and bottlebrush *Acropora*, are positively associated with parrotfish species in the exposed reefs. As corals with a fine structure are primarily found in sites with less wave exposure (Nishihira & Veron, 1995; Nanami et al., 2005; Nanami, 2020), exposed reefs do not inherently have a high coverage of corals with a fine structure. Under this constraint (less coverage of corals with a fine structure), calcium carbonate substratum might be the primary suitable habitat for parrotfishes in exposed reefs. The high structural complexity of calcium carbonate substratum might also provide merits in terms of hydrodynamic benefits, *i.e*., such high complexity provides large holes with less wave exposure and can be used by parrotfishes as sleeping sites.

Species-specific spatial distribution of parrotfish assemblages might be also caused by inter-specific competitive interactions that have been shown for other fish families (*e.g*., Eurich, McCormick & Jones, 2018a, 2018b). Eurich, McCormick & Jones (2018a, 2018b) have shown the aggression among ecological similar species is one of the determinants of spatial distributions of damselfishes. In contrast, inter-specific differences in foraging substrates might be caused by species coexistence among multiple parrotfish species. Nicholson & Clements (2020) have suggested that inter-specific difference in foraging microhabitat utilization enhance the species coexistence among five parrotfish species. Inter-specific competitive exclusions as well as inter-specific microhabitat utilization for foraging should be investigated for parrotfish assemblages in Okinawan region.

The results of the present study also examined the size-related variations in the spatial distribution of parrotfishes. *Chlorurus microrhinos* showed a clear size-related difference in the spatial distribution. Namely, smaller-sized individuals (TL ≤ 30 cm) and larger-sized individuals (TL ≥ 31 cm) were primarily found in the inner reefs and exposed reefs, respectively. This is probably due to the difference in substrate characteristics between the exposed and inner reefs. The inner reefs were characterized by a higher coverage of branching and bottlebrush substrates that had a fine structure, whereas the exposed reefs were characterized by a higher coverage of calcium carbonate substratum and other corals (encrusting and foliose corals) that with a coarse structure. These differences in habitat characteristics might be the main causative factor for the size-specific difference in the spatial distributions of the species. An alternative explanation is that only larger-sized individuals (TL ≥ 31 cm) are suitably adapted to exist in the stronger flows on the exposed reefs.

*Scarus rivulatus* presented a similar size-related difference in the spatial distribution, indicating that smaller-sized individuals (TL ≤ 20 cm) were primarily found in the inner reefs whereas larger-sized individuals (TL ≥ 21 cm) were found in both inner and exposed reefs. This size-related spatial difference might also be related to the habitat structural difference between exposed and inner reefs.

Although *S. hypselopterus* of all size classes was found in the inner reefs, a size-related difference in spatial distribution was found. Smaller-sized and larger-sized individuals were found in sites with a greater coverage of bottlebrush substrates and branching substrates, respectively. As bottlebrush substrates have a finer structure than branching substrates, smaller-sized individuals would show positive association with the substrates with a finer structure.

### Spatial distribution of groupers

Several studies have reported the habitat associations of groupers at a microhabitat level. *Cephalopholis spiloparaea* and *C. urodeta* in the Mariana Islands were positively associated with massive *Porites* corals and rock, respectively (Donaldson, 2002). Juveniles of *Plectropomus leopardus* in the Great Barrier Reef showed positive associations with sand-rubble, rubble mounds, and live corals (Light & Jones, 1997). In contrast, the present study identified the spatial distribution of groupers over a scale of several to tens of kilometers in relation to habitat characteristics. In addition, size-related variations in the spatial variations were also examined, which have been rarely studied in the previous studies. Among seven species, two species (*C. argus* and *C. urodeta*) did not show clear associations with live corals, whereas another two species (*C. miniata* and *Epinephelus ongus*) were found in areas with a higher coverage of live corals with complex structures (bottlebrush and branching *Acropora*). Three species (*P. leopardus*, *V. louti*, and *V. albimarginata*) showed weak associations with live corals. These results indicate that the coverage of live corals did not necessarily have positive associations with the spatial distribution of groupers. Ticzon et al. (2012) showed similar results, indicating that a greater density of *C. argus*, *C. urodeta*, and *V. louti* was found on non-coralline substrates than live corals. Thus, larger-scale habitat characteristics (exposed reefs *vs*. inner reefs) might be better explanatory factor influencing the species-specific spatial distribution of groupers.

Although species-specific spatial variations were found in the seven grouper species, size-related variations in the spatial distributions were not clearly found for all species in the present study (several to tens kilometer scale). This suggests that clear ontogenetic habitat shift would not occur at the landscape-level. Nanami et al. (2013) examined the substrate selection at larval settlement stage for grouper, indicating that pelagic larvae of *E. ongus* selected live corals with complex structures (bottlebrush and branching *Acropora*) at settlement. Since the present study showed the two types of corals were also associated with the larger-sized individuals of *E. ongus*, it is suggested that juvenile settlement might be one of the primary determinants of the spatial distribution of the grouper populations.

As the main prey items of the seven grouper species are fish and crustaceans (Shpigel & Fishelson, 1989; St John, 1999; Nakai, Sano & Kurokura, 2001; Dierking, Williams & Walsh, 2009; Froese & Pauly, 2020), the density of prey items might be one of the main factors determining the spatial distribution of groupers. A higher density of benthic crustaceans was primarily found on non-coralline substrates (dead coral and coral rubble) rather than live corals (Kramer, Bellwood & Bellwood, 2014), and predators of crustaceans (*e.g*., wrasse) also used non-coralline substrates as feeding microhabitats (Kramer, Bellwood & Bellwood, 2016). Some studies have shown that smaller-sized fish, which are potential prey items for groupers (*e.g*., pomacentrids and juveniles of other taxa), are associated with both live corals and non-coralline substrates (Wilson et al., 2008, 2010; Ticzon et al., 2012; Giffin, Rueger & Jones, 2019). These results suggest that foraging sites for the seven grouper species are not restricted to live corals and this might be why the spatial distribution of groupers was not necessarily associated with the coverage of live corals.

## Conclusions

The present study examined the actual spatial distribution of parrotfishes and groupers in an Okinawan region, and suggest that the coverage of live corals are not necessarily the primary factors that are responsible for the species-specific spatial distributions of these two fish groups in the region. However, although both parrotfishes and groupers showed positive associations with substrates with high complexity, it is suggested that the ecological aspect of the association is different. For parrotfishes, it is suggested that high substrate complexity with fine structure would provide refuge space for smaller-sized individuals whereas high substrate complexity with coarse structure would provide resting sites, not refuge spaces, for larger-sized individuals. However, since parrotfishes are herbivores (Nicholson & Clements, 2020), such high substrate complexity might not necessarily affect feeding efficiency. In contrast, high substrate complexity would provide both refuge spaces and foraging places for groupers, since groupers are ambush top predators. Deterioration after coral death would cause the loss of structural complexity (Pratchett et al., 2008). Under the condition, less structural complexity would decrease abundance and species diversity of these two fish groups. Although the present study examined the effects of habitat characteristics on spatial distribution of fishes, other ecological factors (*e.g*., inter- and intra-specific competitions and predation) should be studied as possible effects that control the spatial distributions.

## Supplemental Information

10.7717/peerj.12134/supp-1 Supplemental Information 1 Photographs for 16 substrate types. Note that each photograph represents an example for the each substrate type. The classification of substrate type was based on Pratchett et al. (2015) and Nanami (2018). The photographs of all substrates were taken by the author (A. Nanami).

10.7717/peerj.12134/supp-2 Supplemental Information 2 Total number of individuals for each species during two survey periods (June 2016- January 2017 and June 2017 – February 2018). Average numbers for the two survey periods are indicated. Red lines represent thresholds between dominant and non-dominant species.

10.7717/peerj.12134/supp-3 Supplemental Information 3 Results of the redundancy analysis (RDA) to explain the association between the spatial distribution of the 12 parrotfish species and environmental characteristics. RDA plots for all species as well as all size classes were simultaneously shown as the results of assemblage-level analysis. Environmental characteristics that had significant associations on spatial distributions are presented as blue vectors. Six different colors of symbols (white, sky-blue, yellow, magenta, red, and purple) represent different size classes (see Fig. 2). Some types of substrates are represented with abbreviations (BR Ac: branching *Acropora*; D BR Ac: dead branching *Acropora*; BB Ac: bottlebrush *Acropora*, D BB Ac: dead bottlebrush *Acropora*, CCS: calcium carbonate substratum). The photographs of all fish species were taken by the author (A. Nanami).

10.7717/peerj.12134/supp-4 Supplemental Information 4 Results of the redundancy analysis (RDA) to explain the association between the spatial distribution of the 7 grouper species and environmental characteristics. RDA plots for all species as well as all size classes were simultaneously shown as the results of assemblage-level analysis. Environmental characteristics that had significant associations on spatial distributions are presented as blue vectors. Five different colors of symbols (white, sky-blue, yellow, magenta, and red) represent different size classes (see Fig. 4). Some types of substrates are represented with abbreviations (BR Ac: branching *Acropora*; D BR Ac: dead branching *Acropora*; BB Ac: bottlebrush *Acropora*, D BB Ac: dead bottlebrush *Acropora*, CCS: calcium carbonate substratum). The photographs of all fish species were taken by the author (A. Nanami).

10.7717/peerj.12134/supp-5 Supplemental Information 5 Results of the redundancy analysis (RDA) to explain the relationship between environmental characteristics and site scores. Environmental characteristics that had significant associations on spatial distributions are presented as blue vectors. Some types of substrates are represented with abbreviations [BR Ac: branching *Acropora*; D BR Ac: dead branching *Acropora*; BB Ac: bottlebrush *Acropora*, D BB Ac: dead bottlebrush *Acropora*; BRANCH: branching corals except for *Acropora* (*e.g*., branching *Pocillopora*, *Montipora*, and *Porites*); D BRANCH: dead branching corals; D T Ac: dead tabular *Acropora*; Massive: massive corals (*e.g*., massive *Porites* and Faviidae members); OTHER: other corals (*e.g*., encrusting corals and foliose corals); D OTHER: dead other corals, CCS: calcium carbonate substratum).]

10.7717/peerj.12134/supp-6 Supplemental Information 6 Raw data about spatial distributions of fishes.

## Additional Information and Declarations

### Data Availability

The following information was supplied regarding data availability: The raw data are available in a Supplemental File.
