# Supplementary figures and images for: Spatial distribution of parrotfishes and groupers in an Okinawan coral reef: size-related associations in relation to habitat characteristics
Source: PeerJ. 2021 Sep 3;9:e12134. doi: 10.7717/peerj.12134 (PMC8420873; doi:10.7717/peerj.12134)

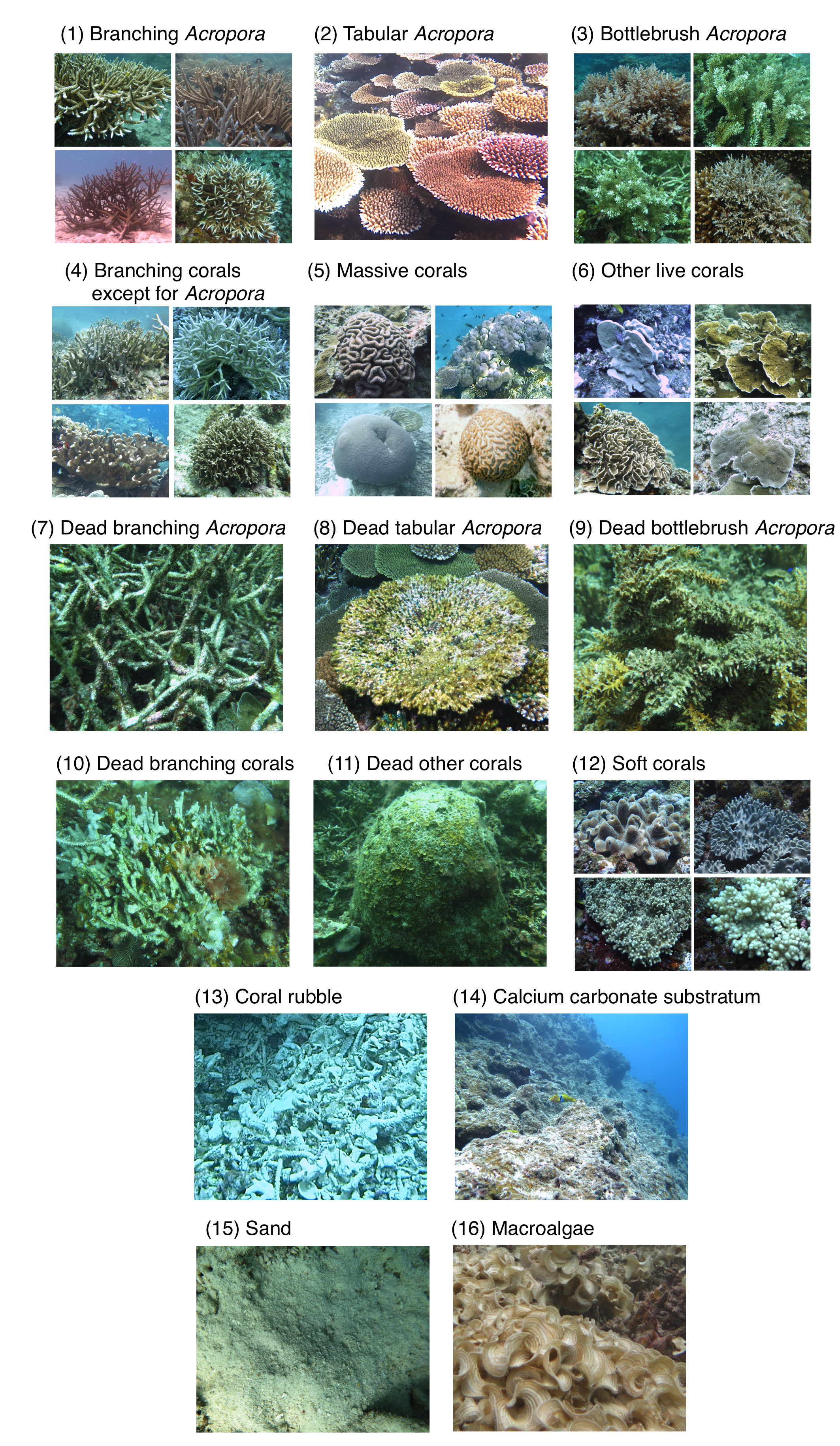

Supplement: Supplemental Information 1 — Note that each photograph represents an example for the each substrate type. The classification of substrate type was based on Pratchett et al. (2015) and Nanami (2018). The photographs of all substrates were taken by the author (A. Nanami). [file peerj-09-12134-s001.png]

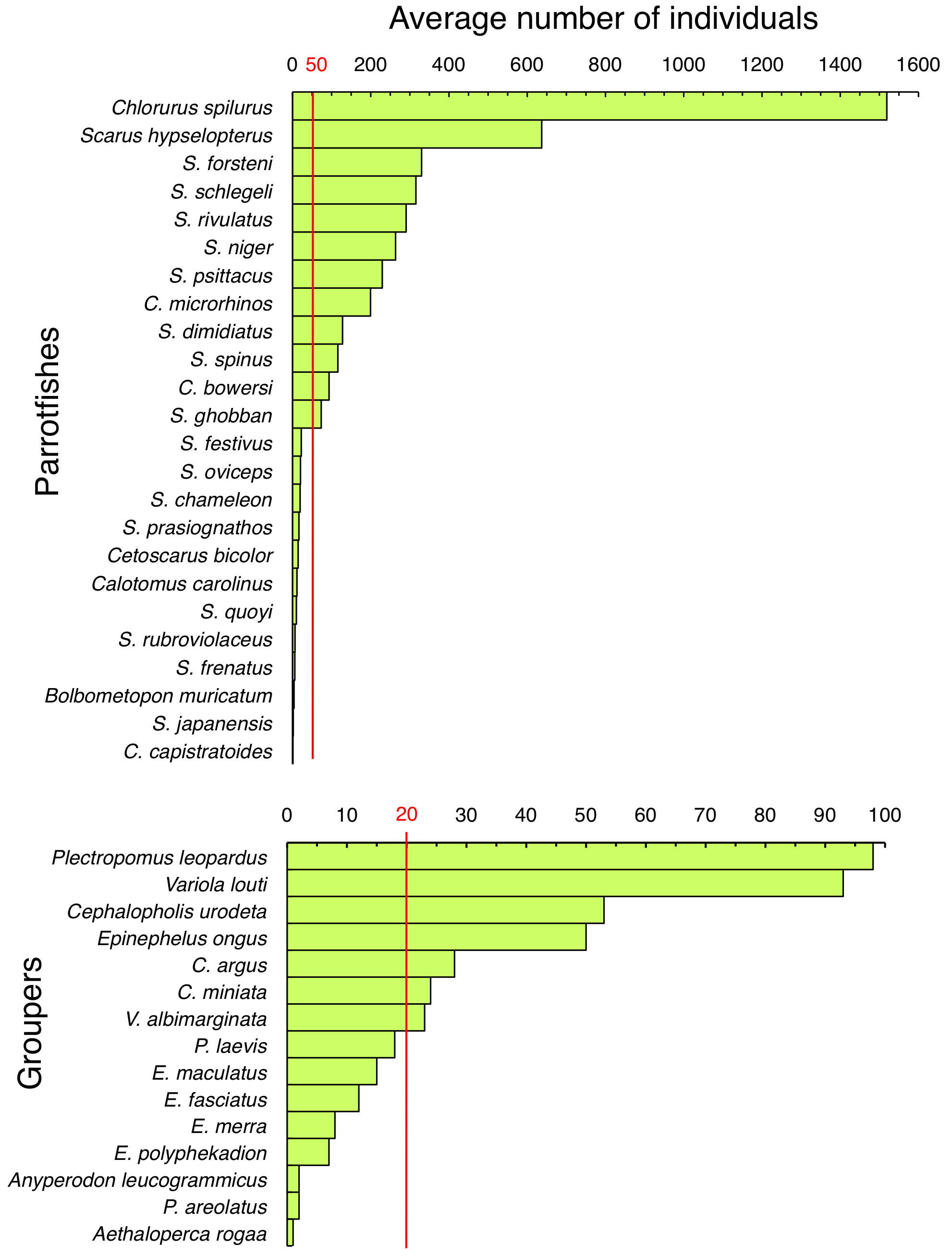

Supplement: Supplemental Information 2 — Average numbers for the two survey periods are indicated. Red lines represent thresholds between dominant and non-dominant species. [file peerj-09-12134-s002.png]

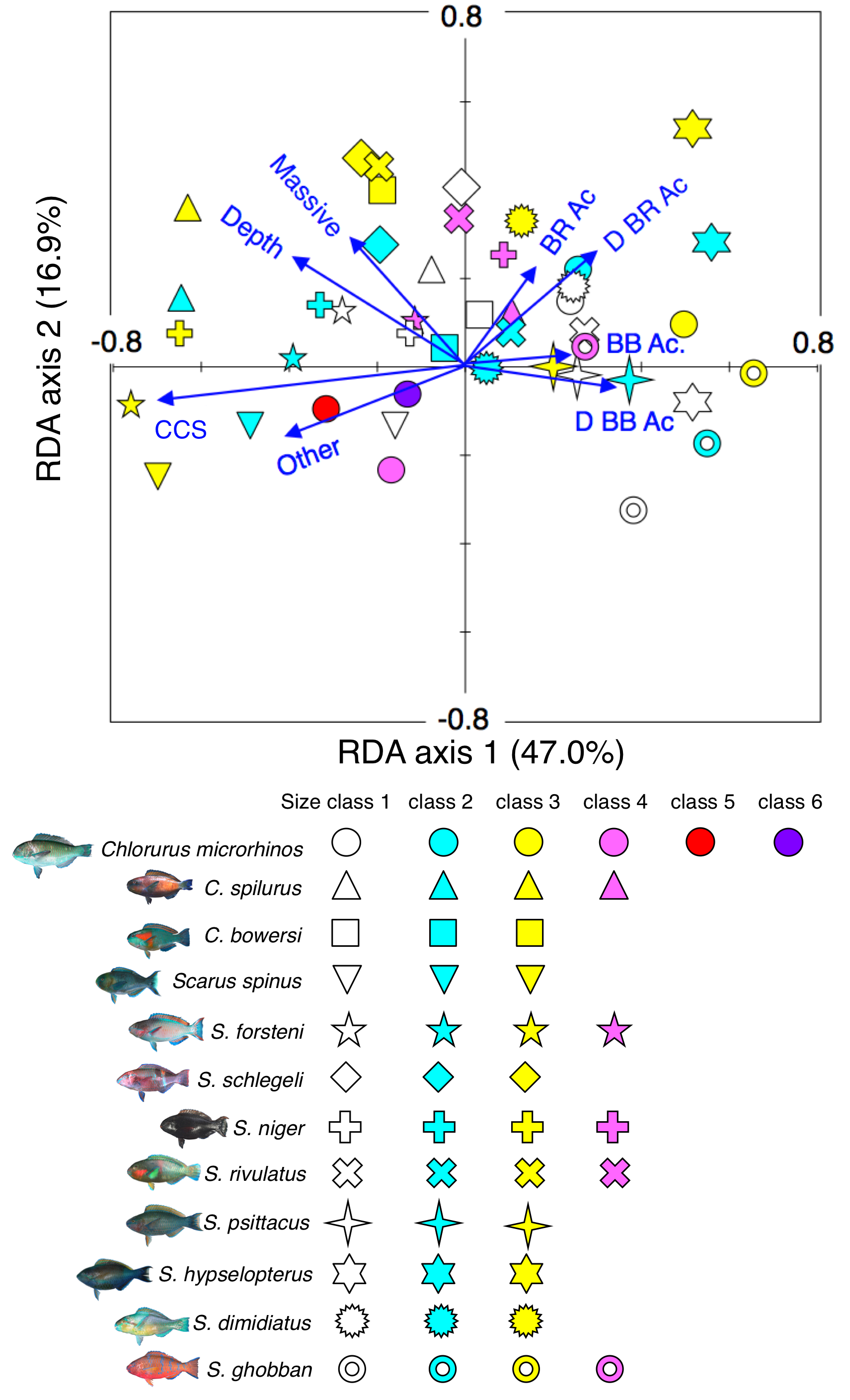

Supplement: Supplemental Information 3 — RDA plots for all species as well as all size classes were simultaneously shown as the results of assemblage-level analysis. Environmental characteristics that had significant associations on spatial distributions are presented as blue vectors. Six different colors of symbols (white, sky-blue, yellow, magenta, red, and purple) represent different size classes (see Fig. 2). Some types of substrates are represented with abbreviations (BR Ac: branching Acropora; D BR Ac: dead branching Acropora; BB Ac: bottlebrush Acropora, D BB Ac: dead bottlebrush Acropora, CCS: calcium carbonate substratum). The photographs of all fish species were taken by the author (A. Nanami). [file peerj-09-12134-s003.png]

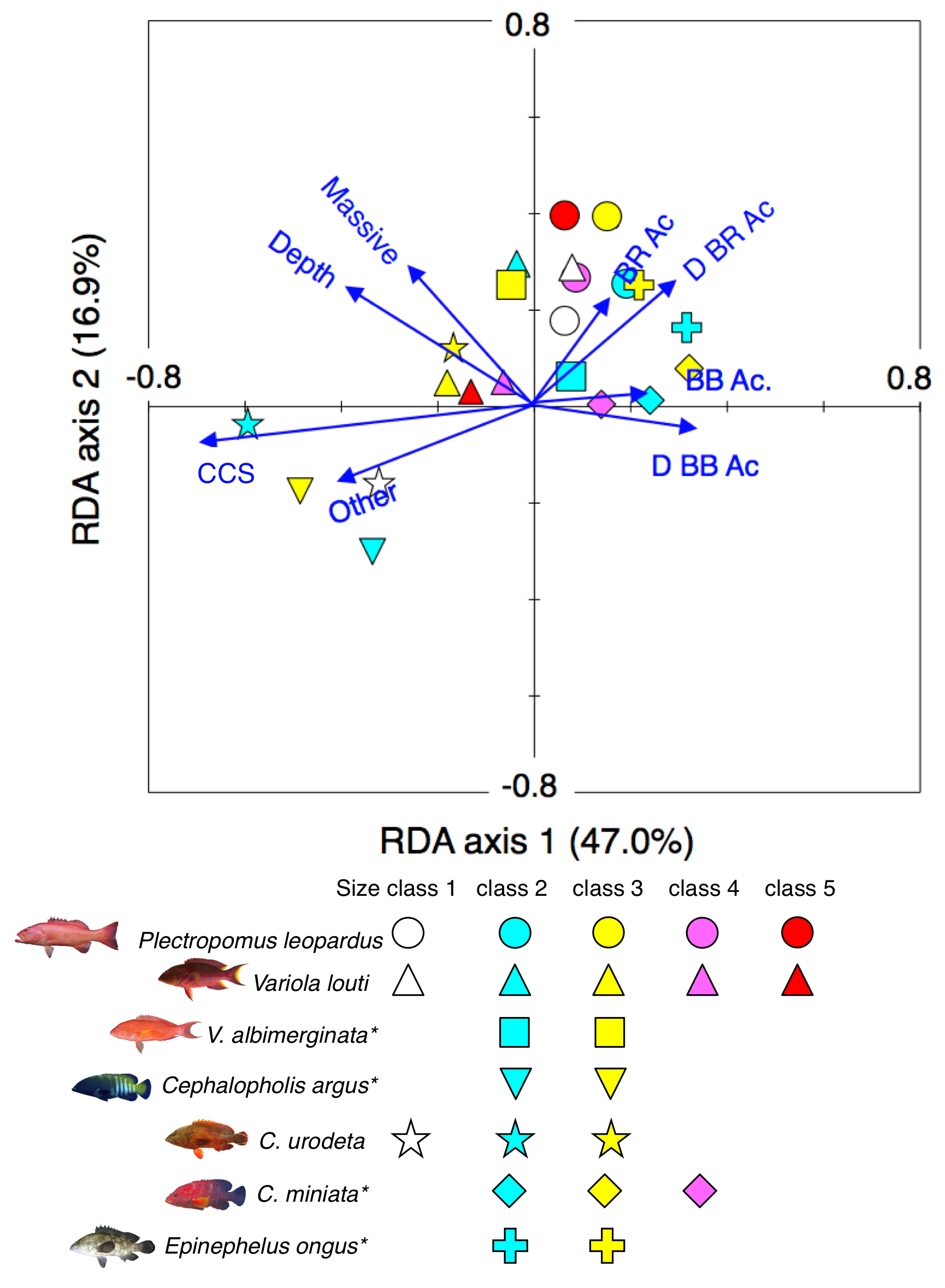

Supplement: Supplemental Information 4 — RDA plots for all species as well as all size classes were simultaneously shown as the results of assemblage-level analysis. Environmental characteristics that had significant associations on spatial distributions are presented as blue vectors. Five different colors of symbols (white, sky-blue, yellow, magenta, and red) represent different size classes (see Fig. 4). Some types of substrates are represented with abbreviations (BR Ac: branching Acropora; D BR Ac: dead branching Acropora; BB Ac: bottlebrush Acropora, D BB Ac: dead bottlebrush Acropora, CCS: calcium carbonate substratum). The photographs of all fish species were taken by the author (A. Nanami). [file peerj-09-12134-s004.png]

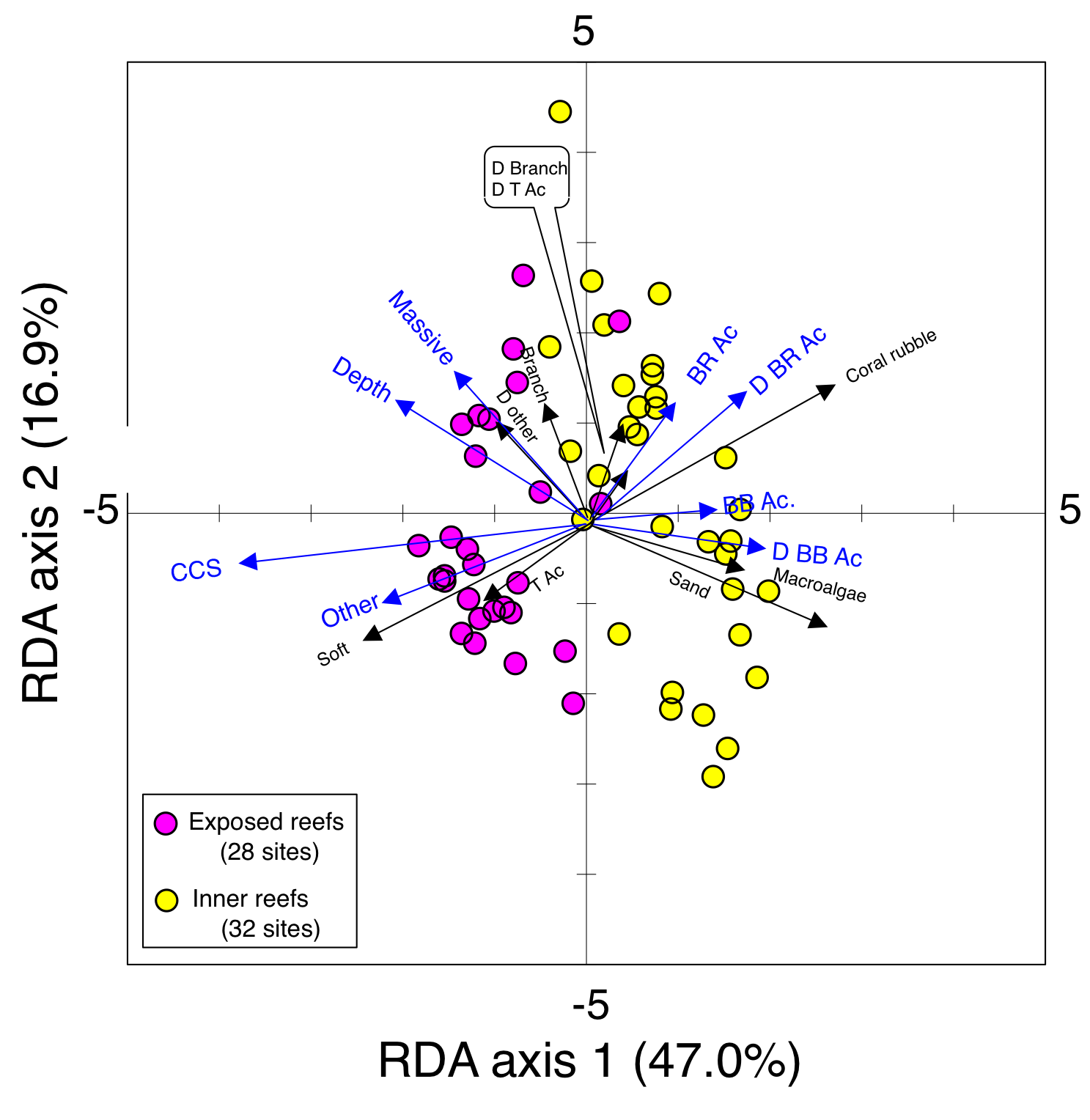

Supplement: Supplemental Information 5 — Environmental characteristics that had significant associations on spatial distributions are presented as blue vectors. Some types of substrates are represented with abbreviations [BR Ac: branching Acropora; D BR Ac: dead branching Acropora; BB Ac: bottlebrush Acropora, D BB Ac: dead bottlebrush Acropora; BRANCH: branching corals except for Acropora (e.g., branching Pocillopora, Montipora, and Porites); D BRANCH: dead branching corals; D T Ac: dead tabular Acropora; Massive: massive corals (e.g., massive Porites and Faviidae members); OTHER: other corals (e.g., encrusting corals and foliose corals); D OTHER: dead other corals, CCS: calcium carbonate substratum).] [file peerj-09-12134-s005.png]
